# Supplementary material for: Natural Catalytic IgGs Hydrolyzing Histones in Schizophrenia: Are They the Link between Humoral Immunity and Inflammation?
Source: Int J Mol Sci. 2020 Sep 30;21(19):7238. doi: 10.3390/ijms21197238 (PMC7582518; doi:10.3390/ijms21197238)
Supplement: Supplementary file 1 [file ijms-21-07238-s001.pdf]

Table S1. Averaged clinical data for schizophrenia patients included in the study.

The number of patients	Diagnosis*	Sex	Age, years	Disease duration, years	Positive and Negative Syndrome Scale (PANSS) Scores**				
					PANSS Positive α<br>1	PANSS Negative β<br>2	Composite index γ	PANSS General Δ<br>3	PANSS Total ε<br>∑1+2+3
**Patients with Leading Positive Symptoms**									
22	F20.00 – 6 patients;<br>F20.01 – 11 patients;<br>F20.02 – 1 patients;<br>F20.09 – 2 patients;<br>F20.6 – 2 patients	11M/ 11F	41,2 ± 11,3	15,0 ± 9,1	20,2 ± 8,3	17,1 ± 7,3	4,2 ± 7,0	40,5 ± 12,6	82,2 ± 25,6
**Patients with Negative Positive Symptoms**									
28	F20.00 – 5 patients;<br>F20.01 – 3 patients;<br>F20.02 – 10 patients;<br>F20.09 – 6 patients;<br>F20.6 – 4 patients	10M/ 18F	37,0 ± 9,5	12,9 ± 7,8	12,1 ± 5,6	19,9 ± 5,1	-7,8 ± 6,7	36,3 ± 8,9	68,6 ± 15,7
**Average values for total group of patients**									
50	F20.00 – 11 patients;<br>F20.01 – 14 patients;<br>F20.02 – 11 patients;<br>F20.09 – 8 patients;<br>F20.6 – 6 patients	21M/ 29F	38,8 ± 10,4	13,8 ± 8,4	15,6 ± 7,9	20,4 ± 6,1	-0,9 ± 7,6	38,1 ± 10,7	74,5 ± 21,4

* In accordance with International Statistical Classification of Diseases and Related Health Problems (ICD-10)

** Symptom severity of patients with schizophrenia was assessed according to "The positive and negative syndrome scale (PANSS)" (Kay, S. R., Fiszbein, A., & Opler, L. A. (1987). The positive and negative syndrome scale (PANSS) for schizophrenia. Schizophrenia bulletin, 13(2), 261-276 [47]).

α PANSS Positive – positive syndrome scale, which refer to an excess of normal mental functions. It includes symptoms such as delirium, thinking disorders, hallucinations, agitation, ideas of greatness, suspicion and ideas of persecution, hostility.

β PANSS Negative – negative syndrome scale, which represent a diminution or loss of normal mental functions. It includes symptoms such as decreased emotionality, emotional fencing, difficulty communicating, decreased initiative, impaired abstract thinking, impaired spontaneity and smooth speech, stereotyped thinking.

γ Composite index – the difference between the PANSS Positive scale scores and PANSS Negative scale scores that reveals the prevailing symptoms.

Δ PANSS General – general psychopathology scale, which assesses the general aspects of psychopathology and the severity of schizophrenia. It includes symptoms such as anxiety, guilt, tension, agility and posing, depression, motor lethargy, complaints of physical health, lack of communication, the presence of eccentric thoughts, disorientation, impaired attention, a breakdown of will, impulsiveness and aggressiveness and others.

ε PANSS Total – total points on PANSS Positive scale, PANSS Negative scale and PANSS General scale.
